# Retraction: Altitude cardiomyopathy is associated with impaired stress electrocardiogram and increased circulating inflammation makers

**DOI:** 10.3389/fphys.2022.1006557

**Published:** 2022-08-04

**Authors:** Frontiers Editorial Office

**Keywords:** treadmill exercise test, acute mountain sickness, simulated hypobaric hypoxia condition, prediction, inflammation

The Publisher retracts the cited article.

Following publication, the publisher uncovered evidence that false identities were used in the peer-review process. The assignment of fake reviewers was confirmed by an investigation, conducted in accordance with Frontiers’ policies and the Committee on Publication Ethics (COPE) guidelines.

The investigation also uncovered concerns about the presentation and validity of the data in the article that would normally have led to a rejection. When contacted, the authors failed to provide a satisfactory explanation. The article is therefore retracted.

The authors do not agree to this retraction.

This retraction was approved by the Chief Editors of Frontiers in Physiology and the Chief Executive Editor of Frontiers.

